# Reply to ‘Concerns about the feasibility of using “precision guided sterile males” to control insects’

**DOI:** 10.1038/s41467-019-11617-8

**Published:** 2019-09-02

**Authors:** Nikolay P. Kandul, Junru Liu, Hector M. Sanchez C, Sean L. Wu, John M. Marshall, Omar S. Akbari

**Affiliations:** 1 Division of Biological Sciences, Section of Cell and Developmental Biology, University of California, San Diego, La Jolla, CA United States of America; 2 Division of Biostatistics and Epidemiology, School of Public Health, University of California, Berkeley, CA United States of America; 3 Tata Institute for Genetics and Society, University of California, San Diego, La Jolla, CA United States of America

**Keywords:** CRISPR-Cas9 genome editing, Genetic engineering

**Replying to** J. Bouyer. *Nature Communications* 10.1038/s41467-019-11616-9 (2019)

We appreciate the comments by Bouyer (2019) on the translation of the precision-guided sterile insect technique (pgSIT), developed in our study for *Drosophila melanogaster*^1^, to other species, in particular *Aedes aegypti*. We acknowledge that a number of assumptions were made in extending the implications of our findings to other species. In this correspondence, we explore the implications of less generous assumptions regarding the field performance of pgSIT in *Ae. aegypti*, and confirm that it is still a competitive technology under these conditions. We also clarify logistical concerns regarding the distribution of eggs, sex sorting of adults, and the potential for mutations to occur that could disrupt field implementation.

Bouyer first notes that, for our simulations of releases of insects carrying a dominant lethal (RIDL) and female-specific RIDL (fsRIDL), the mating competitiveness parameter was taken from the field performance of an *Ae. aegypti* RIDL strain;^2,3^ whereas for pgSIT, it was taken from the lab performance of a *D. melanogaster* strain^1^. We agree that this is an important point that requires further investigation. To this end, in the absence of field data for pgSIT, we have repeated our pgSIT simulations with the mating competitiveness parameter used for RIDL, in which released adult males having the construct had their mating competitiveness reduced by 95% as compared to wild males in the field^2,3^. Results from these simulations suggest a very similar performance between pgSIT and fsRIDL when identical parameters are used (Fig. 1), which is not surprising, as both are self-limiting population suppression technologies that involve releases at the egg stage.

**Fig. 1:**
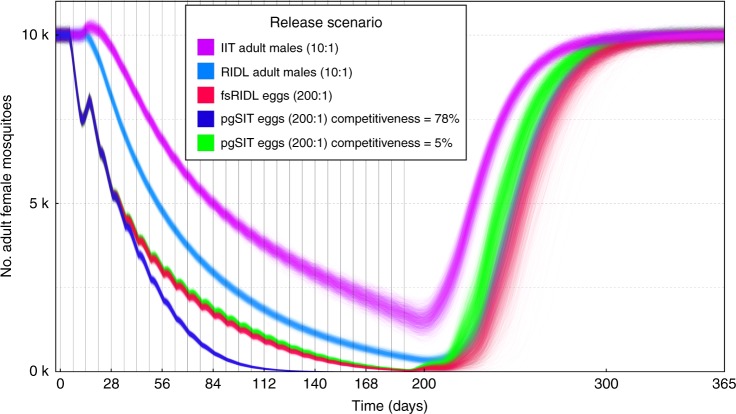
Model-predicted impact of releases of pgSIT eggs on *Aedes aegypti* mosquito population density with comparison to releases of *Wolbachia*-based incompatible insect technique (IIT), release of insects carrying a dominant lethal gene (RIDL), and female-specific RIDL (fsRIDL). Releases are carried out weekly over a 6-month period with release ratios (relative to wild adults) shown in the key. Model predictions were computed using 2000 realizations of the stochastic implementation of the MGDrivE simulation framework^5^ for a randomly mixing *Ae*. *aegypti* population of 10,000 adult females and model parameters described in Supplemental Table 10 of Kandul et al. ^1^ Previous results suggested that pgSIT releases outcompete those of other suppression technologies when pgSIT mating competitiveness is estimated from lab experiments for *Drosophila melanogaster* (purple). However, pgSIT and fsRIDL perform similarly well when mating competitiveness is estimated from field releases of RIDL strains of *Aedes aegypti* (green and red, respectively)

Although we do not have data on the field performance of pgSIT strains of *Ae. aegypti* mosquitoes at this point, several lines of evidence suggest that parameters estimated for the RIDL system may be conservative for the pgSIT system, and hence that pgSIT may have a greater potential to eliminate local *Ae. aegypti* populations than current published versions of the RIDL system. First, we used a conservative estimate of an 18% lifespan reduction associated with the pgSIT construct, based on an estimate for the RIDL system in the lab^4^, despite measuring a lifespan increase associated with the pgSIT system in *D. melanogaster* (Figure 4c of ref. ^1^). The cause of this still needs to be investigated; however, it displays the opposite trend as that seen for RIDL in *Ae. aegypti*. Second, the fitness of RIDL mosquitoes is likely impacted by tetracycline exposure and leaky toxin expression of the dominant lethal gene - two factors that do not affect the pgSIT system. That said, there may be unforeseen fitness consequences of the pgSIT system in *Ae. aegypti*, and as more data is collected in the lab and field, we will update our model projections accordingly.

Second, Bouyer had several questions and concerns regarding the logistics of pgSIT field implementation for *Ae. aegypti*: (i) sex sorting of adults to produce pgSIT eggs, (ii) distribution of eggs in the environment and survival to the adult stage, and (iii) dispersal and mixing of pgSIT adults with wild mosquitoes. First, regarding sex sorting, we believe this not to be an issue given the precent set by the Debug Project of Verily who routinely and efficiently sort female and male mosquitoes with 99.99% accuracy. This readily-available sex-sorting technology will enable the separation of adult females and males from both strains to generate pgSIT eggs in quantities sufficient for wide-scale field implementation. Second, regarding egg distribution and survival, we appreciate Bouyer’s comment regarding the difficulty of distributing eggs to small containers and breeding sites in the environment. We instead envision releases of pgSIT eggs into defined, artificial breeding containers in the field so that mosquitoes may then emerge into the environment with high yield survival from egg to adult. Third, regarding mixing of pgSIT males with wild mosquitoes, these artificial breeding containers would be placed carefully to ensure adequate mixing with the wild mosquito population. If the technology proceeds to field implementation, preliminary ecological assessments would be carried out at field sites, along with mathematical modeling, to design release schemes that would address this concern.

Third, Bouyer highlighted the potential for mutations to occur that could inactivate the Cas9 or gRNA cassettes, as a byproduct of mass rearing millions of insects on a weekly basis. It is true that mutations may occur that inactivate the pgSIT system; however, these are unlikely to be detrimental to field implementation. By comparison, RIDL technology has been successfully implemented in the field despite being likely impacted more frequently by mutations that inactivate the system due to basal expression of the VP16 toxin that ecodes dominant lethality, whereas pgSIT is a binary system and is inactive until crossed. That said, assays for detecting inactive pgSIT mutants would be developed as part of the factory protocol.

Fourth, regarding applications to other species, Bouyer states that releasing eggs is not an option for insect pests such as the fruit fly. This statement is incorrect, as we are actively building pgSIT in the fruit fly *Drosophila suzukii*, a major crop pest that lays eggs in fruit, and have plans to deploy eggs by releasing artificial food substrate in which they have been laid. Finally, in terms of regulatory limitations, we expect pgSIT would be regulated in a similar category to comparable insect technologies (e.g., RIDL), which has been approved for release in many countries and is being used in the field today. In conclusion, we believe that results demonstrated for the pgSIT system in *D. melanogaster* are highly encouraging for application to other species, in particular *Ae. aegypti*, but also including several insect agricultural crop pests. We emphasize the need for further lab and field studies to demonstrate the performance of the system in these species.

## Data Availability

The data supporting the findings of this study are available within the paper and its Supplementary Information files.

## Code availability

Code for generating mathematical modelling will be made upon request.
